# Intraarterial anti-leptin therapy via ICA protects ipsilateral CA1 neurons subjected to ischemia and reperfusion

**DOI:** 10.1371/journal.pone.0261644

**Published:** 2022-01-11

**Authors:** Amit Benbenishty, Jacob Schneiderman

**Affiliations:** 1 Segol School of Neuroscience, Tel Aviv University, Tel Aviv, Israel; 2 Sackler Faculty of Medicine, The Department of Vascular Surgery, Sheba Medical Center, Tel Aviv University, Tel Aviv, Israel; Albany Medical College, UNITED STATES

## Abstract

**Background:**

Brain reperfusion following an ischemic event is essential for tissue viability, however, it also involves processes that promote neuronal cell death. We have recently shown that local expression of the hormone leptin in cardiovascular organs drives deleterious remodeling. As cerebral ischemia-reperfusion (IR) lesions derive expression of both the leptin hormone and its receptor, we hypothesized that blocking leptin activity in the injured brain area will reduce the deleterious effects of IR injury.

**Methods:**

C57BL6 male mice underwent bilateral common carotid artery and external carotid artery ligation. The right hemisphere was reperfused after 12 minutes, followed by intraarterial injection of either a low-dose leptin antagonist or saline solution via the ipsilateral ICA. The left common carotid artery remained ligated. Fifteen IR/leptin antagonist-injected and fourteen IR/saline-injected mice completed the experiment. Five days after surgery brains were collected and samples of the hippocampal CA1 region were analyzed for cell viability (H&E) and apoptosis (TUNEL and caspase3), for neuroinflammation (Iba1), and for signaling pathways of pSTAT3 and pSmad2.

**Results:**

The right hemisphere hippocampal CA1 region subjected to IR and saline injection exhibited increased apoptosis and necrosis of pyramidal cells. Also, increased density of activated microglia/macrophages was evident around the CA1 region. Comparatively, leptin antagonist treatment at reperfusion reduced apoptosis and necrosis of pyramidal cells, as indicated by increased number of viable cells (p < 0.01), and reduced TUNEL (p < 0.001) and caspase3-positive cells (p<0.05). Furthermore, this treatment reduced the density of activated microglia/macrophages (p < 0.001) in the CA1 region. Signaling pathway analysis revealed that while pSTAT3 and pSmad2-positive cells were found surrounding the stratum pyramidal in saline-treated animals, pSTAT3 signal was undetected and pSmad2 was greatly reduced in this territory following leptin antagonist treatment (p < 0.01).

**Conclusions:**

Inhibition of leptin activity in hemispheric IR injury preserved the viability of ipsilateral hippocampal CA1 neurons, likely by preventing apoptosis and local inflammation. These results indicate that intraarterial anti-leptin therapy may have clinical potential in reducing hemispheric brain IR injury.

## Introduction

Stroke is the second leading cause of death globally, rendering 50% of brain ischemic event survivors incapacitated for life. Acute brain ischemia is caused by cerebral artery thrombosis or occlusion by blood borne emboli, comprising 87% of all stroke cases. Noteworthy, stroke exerts a major economic burden on nations worldwide, and annual stroke-related healthcare expenditures in the US exceeds $50 Billion [1]. Throughout the last four decades there has been a growing surge of brain revascularization procedures to reestablish blood flow in the occluded vessels [2]. While essential for saving the ischemic tissue, brain reperfusion also drives deleterious processes such as production of oxygen free radicals and extensive neuroinflammation, which further increases tissue damage beyond the initial ischemia-induced injury, and limits its repair capacity [3]. Therefore, treatments aiming to counteract brain ischemia and reperfusion (IR) injury have been designed to limit local inflammatory response and inhibit apoptotic processes [4].

Leptin is induced by hypoxia [5], and both leptin and leptin receptor (LepR) have been shown to be overexpressed in organs subjected to ischemia, including the heart [6] and the brain [7]. Leptin binds to LepR in the tissue, forming an active complex, which activates the JAK2/STAT3 pathway, thereby promoting apoptosis. Additionally, it has been suggested that overexpression of activated LepR in the brain is associated with extensive neuroinflammation, resulting in neuronal cell death [8].

Upregulated leptin expression in the brain after focal ischemic injury is mostly localized in the penumbra, which is the peripheral tissue layer surrounding the core of severely ischemic necrotic cells [9]. Neuronal cells in the penumbra are still viable, although mechanisms of apoptosis and inflammation have been already put in motion, making them highly susceptible to further damage by reperfusion. Therefore, the main goal of anti-IR therapies is to preserve cell viability in the penumbra.

Leptin is an essential hormone modulating neuroendocrine activities, including food intake and energy expenditure. It plays a key role in innate and acquired immunity, inflammation and hematopoiesis [10]. Leptin was also associated with cardiovascular risk factors such as insulin resistance, obesity, hypertension, and increased incidence of stroke [11,12]. In contrast, multiple *in vivo* studies have demonstrated neuroprotection, reducing brain damage following IR injury. Valerio et al. (2009) [7] administered leptin intraperitoneally to mice prior to permanent focal brain ischemia. This prophylactic leptin therapy reduced cortical infarct size in wild type and in leptin deficient mice (Ob/Ob). Zhang F, et al. (2007) [13] found reduced infarct size and improved neurological behavior in mice subjected to transient MCA occlusion that were treated by intravenous leptin ninety minutes after revascularization. Moreover, leptin therapy administered at reperfusion through intracerebral ventricle infusion promoted neuroprotection in transient brain ischemia [14]. Neuroprotection was also demonstrated in a nonhuman primate model of permanent cerebral ischemia, as systemic leptin administration reduced apoptosis, and diminished infarct size [15]. Mechanistic studies revealed that leptin promotes neuroprotection via activation of NF-kB/c-Rel transcription [7], thereby controlling inflammation, and through activation of the PI3K/Akt signaling pathway [16–18]. Notably, these overwhelming findings of the neuroprotective properties of leptin, were based on systemic administration of the hormone.

In contrast, we have recently found harmful effects of locally expressed leptin in cardiovascular organs. Using aortic aneurysm models in mice we demonstrated that locally expressed leptin in the aortic wall promotes medial degeneration, which leads to aneurysm formation [19]. Subsequently, we succeeded in rescuing aortic wall integrity and prevented the development of aortic aneurysm by counteracting local leptin activity, using peri-aortic application of a potent leptin antagonist [20,21]. In line with these findings, excessive leptin and LepR synthesis detected in human carotid atherosclerotic plaques was associated with local deleterious processes causing lesion instability that promoted clinical symptoms of brain ischemia [22].

Given the overexpression of leptin and lepR in the penumbra of ischemic brain lesions, we hypothesized that blocking the leptin pathway in the tissue site after IR injury with a specific competitive leptin inhibitor, will reduce inflammation and apoptotic processes in the injured region. To this end, we tested the effects of a leptin antagonist (LepA) [23] injected intra-arterially (IA) into the reopened ICA, on hemispheric IR-induced damage. Our study has been focused on the hippocampal CA1 region given its paramount role in proper brain function and cognition, and its increased vulnerability to a variety of metabolic and cytotoxic insults, including IR [24]. The choice of an anti-leptin therapeutic agent to treat IR may seem to be at odds with the leptin’s neuroprotective dogma. However, we demonstrate in the current study that leptin antagonist treatment, administered at reperfusion via intraarterial (IA) injection into the ipsilateral ICA resulted in reduced apoptosis and decreased microglia/macrophages activation, thereby preventing neuronal cell death in the CA1 region.

## Methods

This study was carried out in strict accordance with the recommendations in the Guide for the Care and Use of Laboratory Animals of the National Institutes of Health USA. The protocol was approved by the Committee on the Ethics of Animal Experiments of the Tel Aviv University, Israel (Protocol No. 04-16-064). All surgeries were performed under isoflurane anesthesia, and all efforts were made to minimize suffering.

### Ischemia and reperfusion model

We used a forebrain ischemia model that was generated by a simultaneous bilateral CCA occlusion (BCCAO) and ECA ligation, followed by reperfusion of the right hemisphere 12 minutes after induction of ischemia. We were inspired by the BCCAO forebrain ischemia model published by Onken et al., [25], however, we introduced a few significant modifications. Onken’s approach modeled severe forebrain ischemia, hence, they used profound systemic hypotension, pharmacologically induced by overdose isoflurane to precede BCCAO, thereby inflicting substantial forebrain ischemia, with CBF reduced by 95% of the normal values. We, on the other hand, achieved only 75% reduction of CBF by BCCAO. This enabled us to study a rather subtle, and perhaps more clinically encountered phenomena of brain ischemia, such as middle cerebral artery occlusion (MCA) or carotid artery occlusion in the presence of patent circle of Willis. In our model we also clamped both external carotid arteries (ECAs) to eliminate collateral perfusion from the aortic arch and reduce inter-hemispheric collateral perfusion. For our experiments we used leptin antagonist (LepA), supper-active mouse leptin antagonist (SMLA) (PLR- Rehovot, Israel) [23].

In the current study we used a total of 47 twelve-weeks old wild type C57BL/6 male mice. Seven mice succumbed to intraoperative bleeding and anesthetic complication, and the rest were allocated into three groups: (i) IR/saline-injected (19), (ii) IR/LepA-injected (18) and (iii) 3 non-operated controls. Following surgery 21% died from neurological injury, all within the first 24 hours (5 in saline-injected and 3 in LepA-injected mice). Humane endpoints for euthanasia were defined, including bleeding, lethargy, inability to rise or ambulate, severe dyspnea and seizures. However, none of the animals reached such endpoints. We used only male mice to minimize potential sex-related variability. Female sex hormones influence leptin hormone expression in the brain [26], and modulate the cholinergic system, which affects inflammation and drives metabolic dysfunction [27,28].

**Fig 1:**
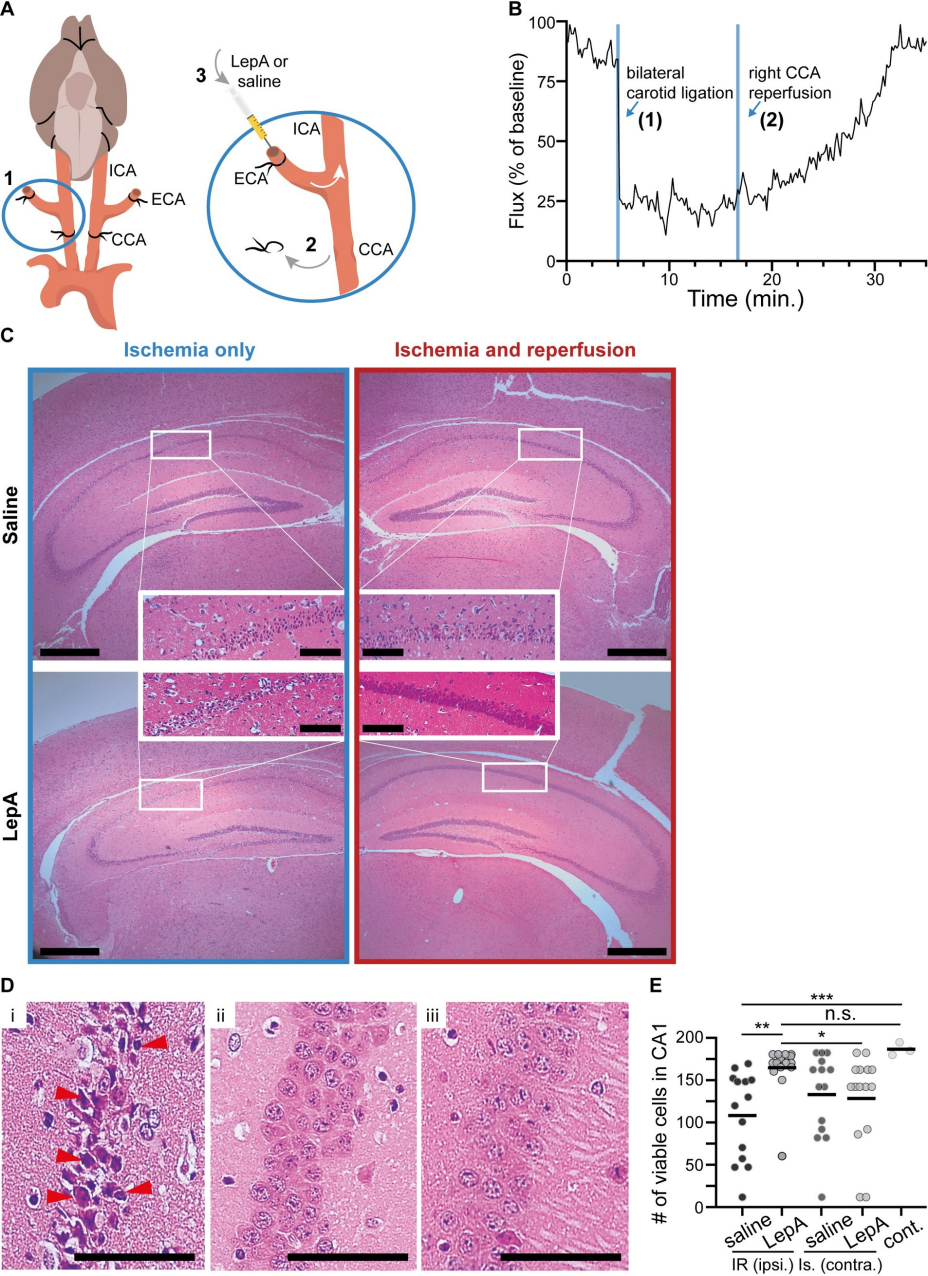
IA LepA injection via ICA limits ipsilateral CA1 cell death following ischemia and reperfusion injury in mice. (**A**) Schematic description of a mouse model to test the effects of targeted LepA treatment on ischemia and reperfusion injury: (1) Bilateral ligation of the common carotid artery (CCA) and external carotid artery (ECA), (2) removal of the ligature from right CCA after 12 minutes of ischemia, (3) concurrent injection at reperfusion using either saline or LepA into the right ICA through the right proximal ECA stump. The left CCA remained ligated throughout the experiment. This model was used to generate right hemispheric IR injury, alongside with simultaneous sustained ischemia in left hemisphere. (**B**) Representative laser doppler flowmetry measurement of the IR model used in the current study. Note the instant drop in flux following bilateral carotid ligation (1), and the gradual increase in flux flowing reperfusion of the right CCA, until reaching baseline levels. (**C**) Representative H&E staining of the hippocampal CA1 region of mice treated with saline or LepA. The right hemisphere was subjected to ischemia and reperfusion, and the left hemisphere was exposed to ischemia only (presented images are from same slide for each treatment group). (**D**) Pyramidal cells in right hippocampal CA1 region, subjected to (i) IR/Saline (ii) IR/LepA (iii) control (*i*.*e*., non-operated). (**E**) Viable cell count in ipsilateral CA1 region proved to be significant (n = 14 in the saline treatment group, n = 15 in the LepA treatment group and n = 3 in the control group, Kruskal-Wallis H = 18.77, *p < 0*.*0001*), with less viable cells in IR/saline compared to IR/LepA (*p = 0*.*0023*) and to control (*p = 0*.*0009*). No differences were found between the ischemia only (i.e., contralateral hemisphere) of saline-treated versus LepA-treated mice (Mann-Whitney U = 96.50, p = 0.7172). Also, in IR/LepA there were more viable cells in the ipsilateral hemisphere than in the contralateral hemisphere (Wilcoxon test, W = -69.00, p = 0.0293), while this was the opposite in the IR/saline group (Wilcoxon test, W = 61.00, p = 0.0563). Data is represented as mean for all data points. Scale bars are 500 μm and 100 μm for low and high magnification, respectively.

For surgery, mice were anesthetized and maintained on 1.5% isoflurane in a 30/70 oxygen/N_2_O mixture; the body temperature was maintained at 37°C, using a warming pad, and monitored body temperature by a rectal probe. Post-procedural care was taken to minimize mouse suffering, assessing animals’ condition daily, and administering analgesia, using carprofen 5mg/kg IP for 3 consecutive days. The surgical procedure included bilateral exposure of the common carotid artery (CCA), proximal internal carotid artery (ICA) and proximal ECA. Both CCAs and ECAs were ligated simultaneously to ensure complete forebrain ischemic conditions, preventing alternative perfusion via collaterals from the contralateral hemisphere and aortic arch. The right CCA ligature was removed after 12 minutes followed by instant intraarterial bolus injection of either leptin antagonist [23] (LepA, 20 μg in a volume of 20 μl) or 0.9% saline (20 μl volume) alone into the right internal carotid artery (ICA) through short ECA stump. For injection, we used the assisted external carotid artery inoculation (aECAi) [29]. Briefly, a fine needle injector with a 34G beveled needle mounted to a micromanipulator (M33, Sutter) was used and manipulated under microscopic guidance (see model scheme, Fig 1A). All animals that completed the study were euthanized after 5 days, using overdose of isoflurane and CO_2_.

### Laser Doppler flowmetry

To assess the impact of the ischemia and reperfusion in our model (Fig 1A), regional cerebral blood flow (rCBF) was measured using laser Doppler flowmetry. Before each measurement was taken, the skull was thinned above the somatosensory cortex (-2 mm from the Bregma and 3 mm lateral) in mice subjected to IR, to a depth below the vessels of the bone, avoiding penetration. Thereafter, a metal custom-made probe holder was attached to the skull over the thinned area using dental cement. rCBF was monitored with a laser Doppler probe (Periflux System 5010, Perimed AB), which was securely screwed to the probe holder to avoid its movement. The Doppler signal was recorded at a sampling rate of 10 kHz, and expressed as percentage of baseline (i.e., average of a 5 min period preceding occlusion procedures).

Bilateral carotid ligation caused an instant and significant (~75%) drop in regional blood flux in the ipsilateral right hemisphere. This reduction in blood flow persisted if the carotids in both sides were ligated (12 minutes). Following reperfusion of the right CCA, blood flow gradually increased until reaching baseline levels after approximately 15–20 minutes (Fig 1B). Based on our experience, slow injection through the ECA stump, as performed herein, does not change regional cortical blood flow [29].

### Tissue analysis

Brains were fixed in paraformaldehyde (4%) and processed for paraffin embedding. All histological analyses were performed on 5μ thick brain slices. Our analysis was focused on the CA1 region in both hippocampi. Hematoxylin and Eosin (H&E) stained slides were used to assess pyramidal cell viability, based on cell prevalence and nuclear morphology, as previously described [30]. Normal, healthy neurons were identified by their rounded and pale nuclei, whereas degenerating neurons had smaller cell bodies and pyknotic nuclei. Thus, cells that exhibited deformed nuclear shape and pyknosis were defined as non-viable. Blinded assessment was performed by two reviewers, and viable cells were counted within a uniform one-millimeter-long frame, applied at, and including the entire width of the CA1, under x20 magnification. One representative slice at the central part of the CA1 was chosen for each mouse (Bregma -2.18-(-2.46) mm). Immunohistochemical analysis used anti-mouse Abs for caspase3 (CST # 9664), Iba-1 (Wako # 019–19741), p-STAT3 (CST **#** 9145) and p-Smad2 (Millipore **#** ab3849). Three slices were analyzed and averaged in each animal. These immunohistochemistry analyses were performed on the Leica Bond automated staining platform. For TUNEL assay we used the *in-situ* cell death detection kit TMR red (Merck, 12156792910 Roche), which detects single- and double-stranded DNA breaks that occur at the early stages of apoptosis. Paraffin-embedded tissue slides were permeabilized and incubated with the TUNEL reaction mixture that contains TdT and TMR-dUTP. During this incubation period, TdT catalyzed the addition of TMR-dUTP at free 3′-OH groups in single- and double-stranded DNA. After washing, the label incorporated at the damaged sites of the DNA was imaged by fluorescence microscopy (Leica TCS SP8 confocal microscope). Only cells demonstrating intranuclear red signal were considered positive, containing degraded DNA. For H&E histology, to determine ischemia and reperfusion damage (Fig 1), we analyzed brain tissue from 15 IR/LepA-treated and 14 IR/saline treated control mice. For immunohistochemical analyses 4–8 samples were used from each treatment group.

Animal experiments were conducted with the assistance of a neurobiology laboratory at Tel Aviv University. Slides were produced, stained, imaged, and analyzed independently in three different labs–Patholab in Nes Ziona Israel, BWH pathology core at Harvard Medical School, Boston MA USA, and at the Hebrew University, Hadassah Medical Center, Jerusalem.

### Statistical analysis

**Fig 2:**
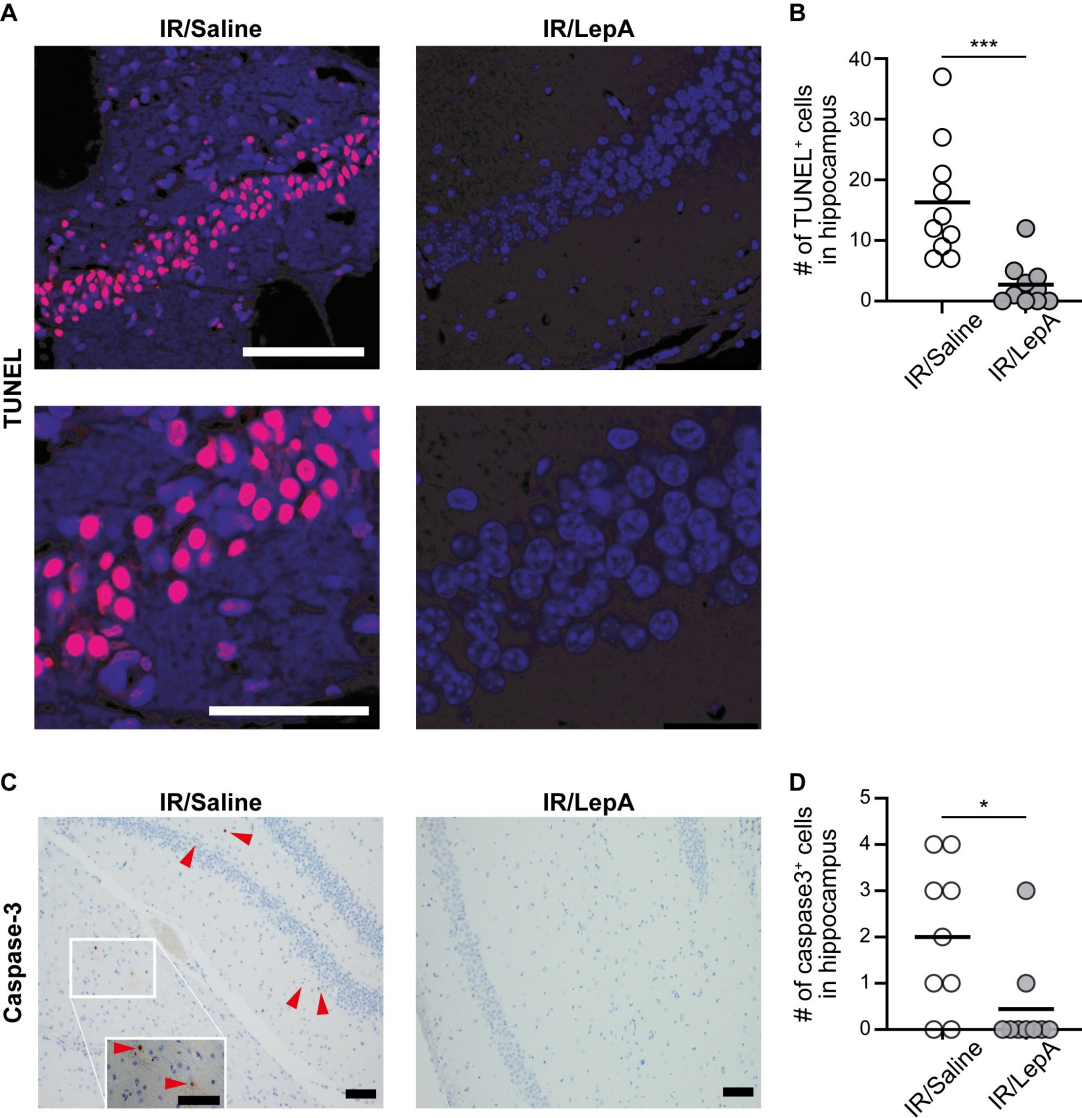
LepA applied IA into ipsilateral ICA at reperfusion prevents apoptosis. (**A**) Representative TUNEL analysis images indicating increased CA1 cell death in saline treated mice versus LepA treated mice. Nuclear signal (magenta) represents fragmented DNA in nucleus (Dapi; blue) of dying cell. (**B**) Less TUNEL staining was evident in following LepA treatment compared to saline (n = 10, unpaired two-tailed Mann-Whitney U = 4.5, *p = 0*.*0002*). (**C**) Representative images of caspase3 staining following ischemia and reperfusion. Caspase3 was evident in (1) CA1 region of IR/saline-injected mice (arrows), but not in (2) IR/LepA-injected counterparts. (**D**) Less caspase3 positive cells were evident in the IR/saline group compared to IR/LepA group (n = 9, unpaired two-tailed Student t-test, t_(16)_ = 2.485, *p = 0*.*0244*). Scale bars are 100 μm.

**Fig 3:**
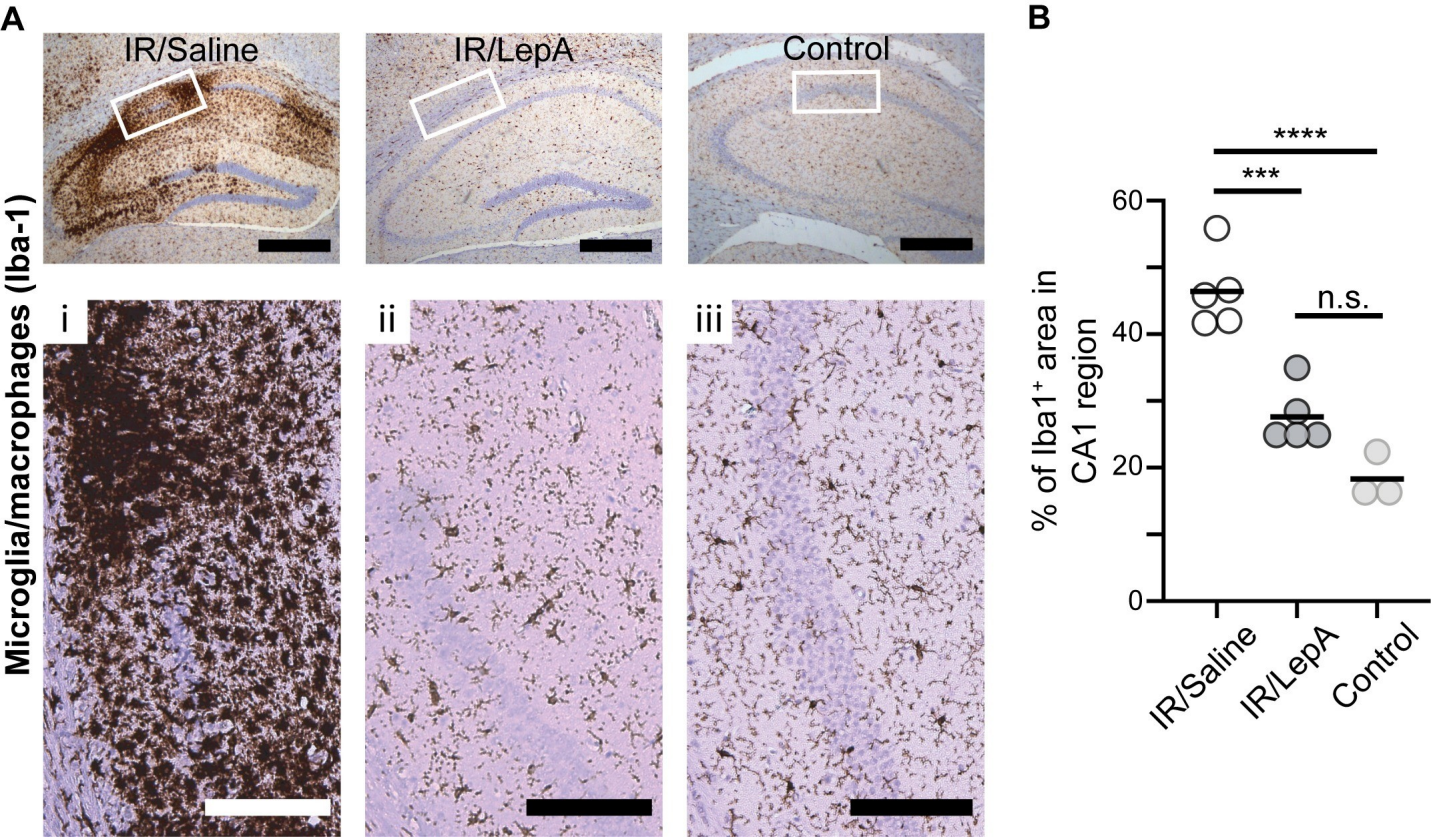
Intraarterial LepA injection via ipsilateral ICA following ischemia and reperfusion injury reduces neuroinflammation in the CA1 region. (**A**) Activated microglia are abundant in the CA1 region of IR/saline-injected samples (i), fewer in IR/LepA-injected samples (ii), and mildly less in control (untreated mice; iii). Scale bars are 500μm and 200μm for low and high magnification, respectively. (**B**) IR resulted in changes in Iba-1 density (n = 8 for saline and control groups and n = 13 for LepA group, Kruskal-Wallis H = 22.89, *p < 0*.*0001*), with increased density of Iba1-positive cells in the CA1 region of IR-saline mice compared to IR-LepA (*p* = *0*.*0127*) and control, untreated, mice (*p < 0*.*0001*). A small increase in Iba-1 was evident following IR with LepA treatment compared to control (*p = 0*.*0420*).

All measured data are presented as means with all analyzed data points. When appropriate, the Kolmogorov-Smirnov normality test was used to determine normal distribution of the data, and the F0test or Brown-Forsythe test to determine homogeneity of variance. For normally distributed data with equal variance, we used two-tailed unpaired Student t-test (Fig 2D) to compare experimental groups. For normally distributed data with unequal variance, we used non-parametric Mann-Whitney U (Figs 1E and 2B), Wilcoxon (Fig 1E), or Kruskal-Wallis (Figs 1E and 3B) tests to compare experimental groups. For post hoc analysis (Figs 1E and 3B), multiple comparisons were corrected using Dunn’s test. P-value of p < 0.05 was considered statistically significant. We used Prism (version 8) for analyses. In all experiments, measurements were taken from distinct samples (i.e., different animals). All data used to generate figure panels can be found in the supplementary information (**S1 Data**).

## Results

### Intraarterial LepA injection via ICA at reperfusion limits ipsilateral CA1 cells’ death following IR injury

We tested the effects of LepA treatment injected into the right ICA at reperfusion after 12 minutes of ischemia (the experimental design is illustrated in Fig 1A). LepA treatment significantly reduced neuronal damage in the hippocampal CA1 regions in the hemisphere that underwent IR, compared to saline injection (*p < 0*.*001*; Fig 1C–1E). Specifically, in the IR/saline group, CA1 neurons exhibited nuclear pyknosis and cytoplasm shrinkage, while in the IR/Lep group they were largely preserved and morphologically resembled neurons in the control non-operated mice (Fig 1C and 1D). Although we focused our analyses on the hemisphere that underwent ischemia and reperfusion, we also counted viable cells in the CA1 region of the contralateral hemisphere that endured ischemia only for 5 days. Pyramidal cell viability in the CA1 region of IR/LepA treated hemisphere was significantly higher compared to the ischemia only contralateral hemisphere (p<0.05). In contrast, in the IR/saline treated group the trend suggested lower cell viability in the ispsilateral compared to the contralateral hemisphere (p = 0.056). Additionally, we found no differences in cell viability between the ischemic hemispheres of LepA and saline treated animals (p > 0.5). Together, these findings demonstrate a targeted and confined effect in the ipsilateral hemisphere CA1 region, attributable to LepA administration.

### Intraarterial LepA injected via ICA at reperfusion attenuates apoptosis in the ipsilateral CA1 region

To further assess the degree of apoptosis we performed TUNEL assay, which demonstrates DNA fragmentations, and analyzed expression of caspase3. We found significantly more TUNEL-positive cells in the CA1 region of IR/saline compared to LepA-treated IR (*p < 0*.*001*; Fig 2A and 2B). These results were in line with extensive positive staining for caspase3 in IR/saline mice, which was largely absent in IR/LepA samples (*p < 0*.*05*; Fig 2C and 2D).

### Intraarterial LepA injection via ipsilateral ICA attenuates neuroinflammation in the CA1 region after IR

To evaluate the degree of inflammation we examined microglia/macrophages Iba-1 expression. We found considerably more Iba-1 positive microglia/macrophages in IR/saline compared to non-operated mice (*p < 0*.*0001*; Fig 3). While Iba-1 staining was also higher in IR/LepA mice compared to non-operated mice (*p = 0*.*0420*) in the stratum pyramidale, it was significantly lower compared to IR/saline mice (*p = 0*.*0127*) with a “non-activated state” morphology (i.e. reduced soma size and finer processes), indicating LepA reduced local inflammation.

### Signaling pathways underlying the response to intraarterial LepA therapy via ipsilateral ICA, promoting rescue of neuronal cell viability

**Fig 4:**
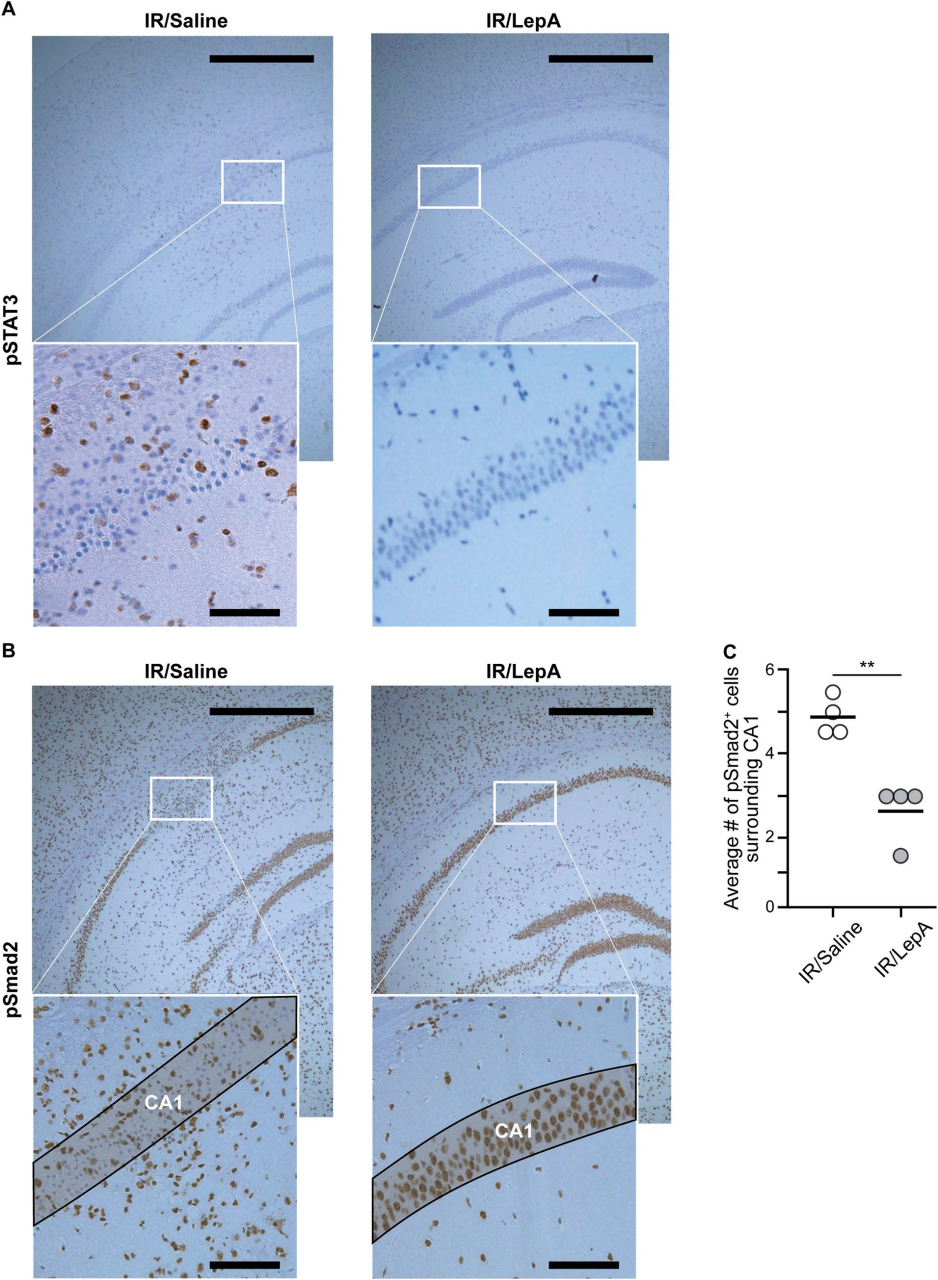
Intraarterial LepA injection via ipsilateral ICA reduced pSTAT3 and pSmad2 levels in the CA1 region following ischemia and reperfusion. (**A**) Representative images of pSTAT3 signal indicating its presents in IR/saline-injected (brown), but not in IR/LepA-treated mice. (**B**) Representative images of pSmad2 signal demonstrated that all viable pyramidal cells in both treatment groups were pSmad2 positive, while in the surrounding of the CA1 region pSmad2 was higher in IR/saline-injected mice compared to IR/LepA-injected counterparts. Scale bars are 500 μm and 100 μm for low and high magnification, respectively. (**C**) Less pSmad2-positive cells were found surrounding the CA1 region in IR/saline compared to IR/LepA treated mice (n = 4, unpaired two-tailed Student t-test, t_(6)_ = 5.058, *p = 0*.*0023*).

To investigate mechanisms leading to apoptosis and inflammation, we stained for the pSTAT3 and the pSmad2 pathways. We found scattered pSTAT3-positive cells in the CA1 region of IR/saline-injected samples, but none in IR/LepA-treated samples (Fig 4A). In contrast, pSmad2 staining was evident in viable neuronal cells of the stratum pyramidale in both treatment groups. However, surrounding the CA1, pSmad2 signal was more prevalent in IR/saline-injected samples than in those treated with IR/LepA (*p < 0*.*01*; Fig 4B and 4C).

## Discussion

In this study we tested the protective efficacy of leptin antagonist (LepA) against cerebral IR injury, through competitive inhibition of leptin in the forebrain of the hemisphere that was subjected to IR injury. LepA was administered intraarterially into the ipsilateral ICA, which supplies the forebrain of the right hemisphere. We found that LepA treatment at reperfusion preserved the viability of ipsilateral hippocampal CA1 neurons.

Our decision to block leptin at the site of injury was motivated by previous observations indicating that locally synthesized leptin in cardiovascular tissues, like the aorta, exerts local deleterious effects via paracrine pathways, thereby promoting medial degeneration and aneurysmal dilatation [19]. Using models of aortic aneurysm in AngII-infused ApoE deficient mice, and in Marfan-like mice we found inhibition of aortic wall degeneration by peri-aortic LepA application in the ascending aorta. Peri-aortic local application of low dose, slow-release LepA that was attached to the wall segment overexpressing leptin pacified local inflammation and prevented MMP-9 expression, consequently preserving cellular viability and vessel wall integrity [20,21].

In the current study we have modified our local therapeutic approach and injected low-dose LepA into the artery feeding the injured tissue, thereby providing regional treatment, rather than systemic dissemination of the antagonist. A regional impact of the drug was most likely obtained due to the high binding affinity of LepA to LepR, which is approximately 60-fold higher than that of endogenous leptin [23]. Thus, intraarterial application of LepA through the ipsilateral ICA, in proximity to the target tissue, along the immediate outflow of the receiving vessel, facilitated rapid binding of most of the LepA to local leptin receptors. As IR results in immediate increase in BBB permeability [31], the drug should have no problem to penetrate locally disrupted BBB and have access to the tissue. Furthermore, although we lack information about LepA capabilities to cross the BBB, an earlier version of leptin antagonist that was injected into the blood stream was shown to penetrate the brain [32]. Notably, the hippocampus receives its arterial blood supply from a capillary network originating in the posterior cerebral artery, which is primarily supplied by the ICA [33]. Therefore, as the antagonist is administered into the ICA by bolus injection, it has a rapid access to the hippocampal tissue. Notably, we have previously used this IA inoculation approach for targeting tumor cells into the brain and proved that albeit their need to cross the blood-brain barrier (BBB) to infiltrate the brain, more cells were found in the cranium than in other organs [29].

The exclusive neuroprotective effects in the ipsilateral hemisphere versus injury in the unprotected contralateral hemisphere highlight the regional nature of our therapeutic approach. Interestingly, in a previous experiment testing leptin-related myocardial IR injury in mice we demonstrated that systemic administration of LepA in the presence of IR injury augmented leptin and LepR expression in the heart and in various other organs instead of blocking them, probably due to activation of compensatory mechanisms [34]. However, in the context of the current experiment we did not anticipate any meaningful LepA leakage to the peripheral circulation.

**Fig 5:**
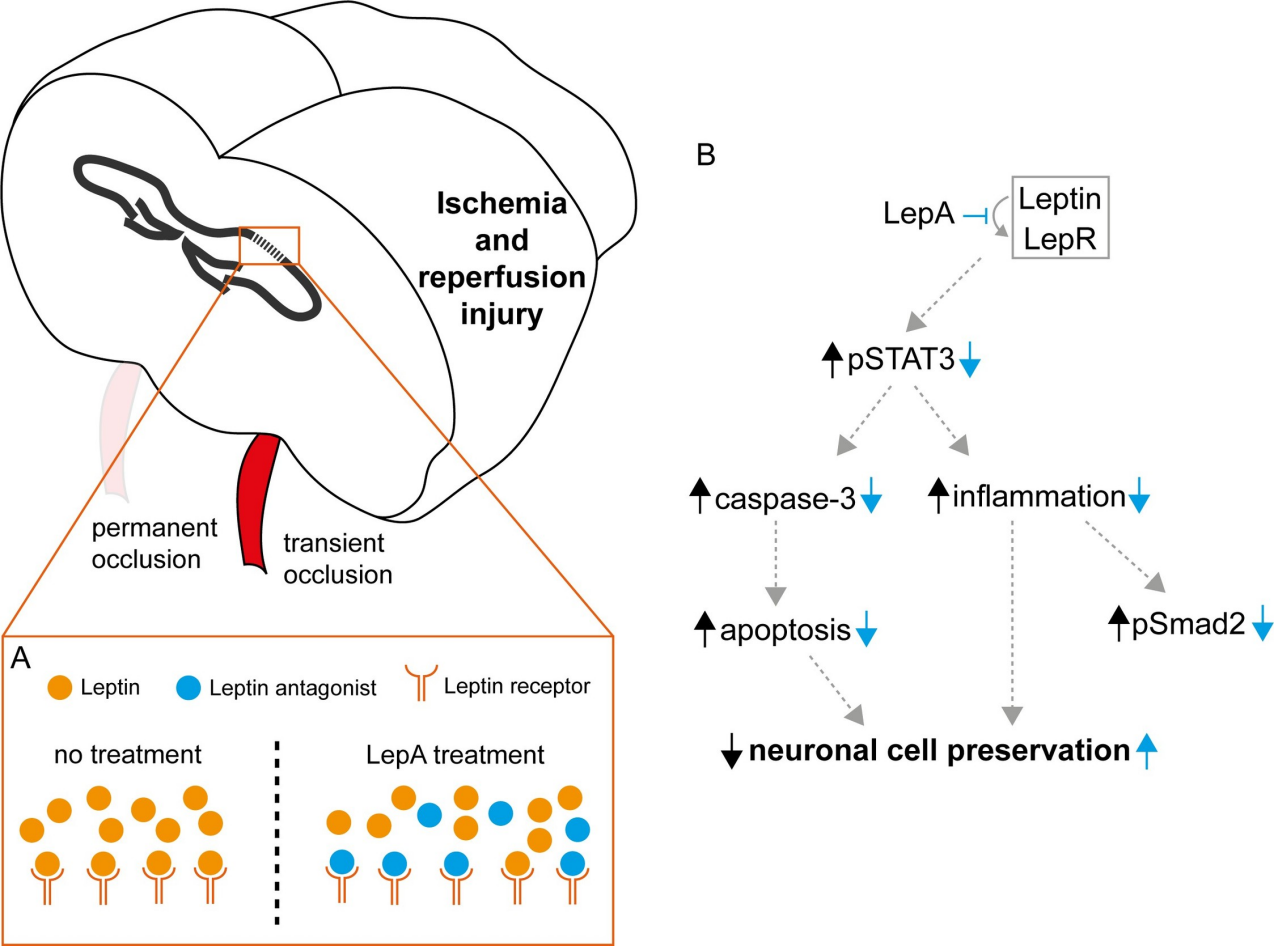
Schematic representation of signaling pathways pSTAT3 and pSmad2 effects in the CA1 region after IR injury, in response to LepA IA injection via ipsilateral ICA at reperfusion. (**A**) Without treatment, leptin overexpression induced by IR injury attaches to upregulated LepRs to form an active complex, while following LepA IA treatment most LepRs are occupied by LepA molecules. (**B**) Without LepA treatment the leptin-LepR complex (black arrows) induces pSTAT3 upregulation and subsequent caspase3 expression (apoptosis) and inflammation. The outcome of this cascade is increased cell death. However, LepA treatment (blue arrows), prevents leptin-LepR complex formation, resulting in reduced pSTAT3, caspase3, and inflammation. Subsequently, the outcome of LepA treatment at reperfusion is neuronal cell preservation.

Studies in focal brain ischemia in mice revealed *de novo* synthesis of leptin and LepR in the penumbra of the ischemic lesions [7]. Together, leptin and LepR form a complex that activates the JAK2/STAT3 signaling pathway, which promotes local apoptosis and accumulation of immune cells, including activated microglia and macrophages, in first few days of injury. These cells express proinflammatory cytokines such as IL-6, IL-1β, TNF**β** and nitrous oxide, which further the neuronal damage [35–37]. Here we also found increased damage to pyramidal cells in the CA1 region of unprotected IR/saline-injected hemispheres (Fig 1) that was associated with upregulated pSTAT3 (Fig 4A), increased apoptosis (Fig 2), and high concentration of activated microglia/macrophages in the area surrounding the stratum pyramidale (Fig 3). Furthermore, data from a rat model of focal brain ischemia revealed that intracerbroventricular infusion of a pJAK2 inhibitor, an upstream regulator of STAT3 has reduced infarct size, downregulated apoptosis and moderated the severity of neurological damage. In line with these findings, we show in our study that injecting low dose LepA bolus at reperfusion into the feeding vessel of the injured territory, reduced pSTAT3 expression around the ipsilateral CA1 region, and achieved local neuroprotection. Ischemia and reperfusion have been previously shown to activate microglia in the CA1 region [38,39]. With respect to this outcome, we show that neuroinflammation was hindered locally by LepA, and that the decreased density of microglia/macrophages (i.e., Iba1) was associated with rescue of pyramidal cells in the CA1 region (presented schematically in Fig 5).

In contrast to the STAT3 pathway, the Smad2/3 pathway acts as a neuroprotective mechanism in focal brain IR injury. Previous experiments in rats exhibited upregulated Smad2/3 in the penumbra following ischemia/reperfusion, and those effects negatively correlated with cell apoptosis [40]. That study also demonstrated that activation of the Smad2/3 pathway in IR lesions downregulates gene expression of inflammatory cytokines and reducing caspase-3 and DNA fragmentation, while increasing the expression of anti-apoptotic protein Bcl_2_. Our results demonstrated IR-induced upregulated expression of pSmad2 in viable pyramidal cells as well as in microglia/macrophages surrounding the CA1 region in both treatment groups. However, reduced inflammation in LepA-treated mice was associated with fewer pSmad2 positive inflammatory cells. Therefore, we suggest that the neuroprotective effects of pSmad2 in our study were most likely mitigated in the LepA treatment group, while downregulation of pSTAT3 by LepA via reduced leptin activity played a dominant role in preserving the viability of neuronal cells in this group.

Our previous studies on aortic aneurysms uncovered a potent anti-inflammatory response to locally applied LepA. In the current research we found similar paracrine-like regional effects in the brain when using an IA LepA injection. These results emphasize the distinction between our IA approach and systemic leptin administration, as the latter was shown to have opposite effects. Specifically, systemic leptin was shown to affect innate immunity by enhancing cytotoxicity of natural killer (NK) cells, and to increase the attraction and activation of granulocytes, macrophages, and dendritic cells, thereby augmenting production of proinflammatory cytokines. Furthermore, leptin stimulates adaptive immunity by promoting T cells and B cells proliferation and decreasing the proliferation of regulatory T (Treg) cells [10].

Human data indicate that hyperleptinemia is associated with various manifestations of cardiovascular disease, including leptin resistance, obesity, insulin resistance and diabetes mellitus, arterial hypertension, coronary disease and increased incidence of stroke [11,12,41,42]. A few clinical studies presented circumstantial association between hyperleptinemia and stroke, supposedly through leptin-induced NF-kB activation and consequent local inflammatory response [16]. However, a causal relationship between elevated leptin levels in the circulation and ischemic stroke events is still debatable [18]. Indeed, we previously presented clinical results demonstrating that systemic leptin levels in symptomatic patients who sustained cerebral ischemic events were similar to leptin levels in neurologically asymptomatic patients [22]. In fact, a large body of experimental data suggests that systemic leptin administration is not detrimental, but rather neuroprotective in the context of brain ischemia and IR injury. These studies show that systemic leptin administration had evidently similar effects on the injured tissue as those observed herein by IA injection of the antagonist, rather than the hormone itself. For example, when leptin was administered to gerbils undergoing bilateral transient CCA occlusion, it was shown to have neuroprotective effects in the hippocampus as indicated by histological analyses five days following IR induction [43]. In that study, unprotected control animals exhibited Iba-1 positive-cells accumulation and a strong LepR immunoreactivity surrounding the stratum pyramidale in the hippocampal CA1 region. In contrast, leptin-treated subjects exhibited scarce glial cells and no LepR immunoreactivity in the same area. These results are very similar to those obtained herein when injecting the antagonist to leptin via the ICA, namely, the outcome of both is impeding the formation of an active leptin-LepR complex.

In summary, our results suggest that IA administration of low-dose LepA into the revascularized vessel at reperfusion may become an important adjunct to supplement reperfusion procedures when treating an ischemic stroke. Moreover, while systemic leptin therapy may require a high dose that likely will result in adverse pharmacokinetics and hormonal perturbations [18], our novel approach is clinically implementable, achieving regional results, and short of systemic complication.

Notably, the current study did not examine the effects of LepA treatment in focal IR injuries however, we demonstrated salvage of pyramidal cells in the CA1 region of the ipsilateral hemisphere, which like the penumbra in focal IR lesions reflects vulnerability and high susceptibility to reperfusion damage. To be considered for clinical implementation the therapeutic efficacy of IA LepA injection should be tested in a brain model of focal IR injury, which represents a more clinically realistic scenario.

Furthermore, in a broader view, microglial activation following ischemia and reperfusion has been suggested to promote neurodegenerative pathologies [44,45]. Therefore, intraarterial LepA therapy administered at reperfusion to the affected territory may blunt this ominous late outcome.

### Limitations of the study

In the current study we used a BCCAO forebrain ischemia model followed by single carotid artery reperfusion to achieve a single hemispheric IR model. An IR model in mice has several limitations and the extent of injury is dependent on mouse strain, time of occlusion, and variability between individual mice, especially regarding the circle of Willis and collateral originating in the aortic arch [46]. However, in our hands this model performed in C57BL/6 mice was reproducible. Notably, our study was focused on the CA1 region of the hippocampus because of its functional importance and susceptibility to IR injury but other cortical and sub-cortical areas of high importance, which most likely have been affected, should be explored, and warrant further investigation.

Additionally, while we clearly show that our therapy improves neuronal cell preservation in the CA1 region, an area important for various cognitive tasks such as learning and memory, we did not study the neurological status, as well as behavioral and cognitive benefits of LepA treatment.

Finally, we focused our analyses on a single time point, namely five days following reperfusion. Our objective was to investigate the acute impact of IR injury before repair processes take place. Therefore, earlier and long-term events at the cell/tissue level, which may have been affected by LepA treatment, need to be investigated in future studies.

## Supporting information

S1 Checklist (DOCX)

S1 Data Aggregated data used to generate figure panels. (XLSX)

## Abbreviations

AngII: angiotensin I
CA1: cornu ammonis 1
CCA: common carotid artery
ECA: external carotid artery
Iba1: ionized calcium binding adaptor molecule 1
ICA: internal carotid artery
IR: ischemia and reperfusion
LepA: leptin antagonist
LepR: leptin receptor
MMP-9: matrix metalloproteinase 9
pSmad2: phosphorylated small mothers against decapentaplegic 2
pSTAT3: phosphorylated signal transducer and activator of transcription 3
TNF**α**: tumour necrosis factor alpha
TUNEL: terminal deoxynucleotidyl transferase biotin-dUTP nick end labeling
