# Supplementary material for: Intraarterial anti-leptin therapy via ICA protects ipsilateral CA1 neurons subjected to ischemia and reperfusion
Source: PLoS One. 2022 Jan 11;17(1):e0261644. doi: 10.1371/journal.pone.0261644 (PMC8752009; doi:10.1371/journal.pone.0261644)
Supplement: S1 Checklist — (DOCX) [file pone.0261644.s001.docx]

**12.03.2021**

**Arrive – Check list**  PONE-D-20-30448R1

**1.Study design**

Focal brain lesions of ischemia and reperfusion (IR) express both leptin hormone and its receptor. We hypothesized that counteracting local leptin activity in hemispheral tissue subjected to IR injury may preserve neuronal viability. The prime objective of this study was to investigate the impact of locally applied leptin antagonist (LepA) on viability of ipsilateral CA1 region pyramidal cells that were exposed to IR.

We generated a forebrain ischemia model in mice by ligating both CCAs and ECAs. To obtain hemispheral IR injury the right CCA was reperfused after 12 min ischemia, and a bolus of low dose LepA or saline solution was instantly injected into the right ICA, at reperfusion. Meanwhile, the left CCA remained ligated to keeping the hemisphere ischemic throughout the 5 days duration of the experiment.

**2.Sample size**

In this study we used 47 mice, 7 of which succumbed to intraoperative bleeding or anesthetic complication. Subsequently, 40 mice were allocated into three groups: (i) IR/saline-injected (19), (ii) IR/LepA-injected (18) and (iii) non-operated controls (3). Amongst the mice undergoing surgery, death related to neurological injury occurred in 21%, all within the first 24 hours (5 in saline-injected and 3 in LepA-injected mice). Additional 7 mice were used for measuring laser doppler flowmetry to demonstrate the impact of bilateral carotid artery ligation and right CCA reperfusion on cortical blood flow in the ipsilateral hemisphere that was subjected to IR injury.

**3. Inclusion and exclusion criteria**

All 29 mice exposed to IR injury that survived beyond 24 hours following surgery were carefully monitored throughout 5 days of follow up and were found to be alert and active. No mouse met criteria for humane euthanasia, including bleeding, lethargy, and inability to rise or ambulate, severe dyspnea or seizures.

**4. Randomization**

All mice underwent a similar anesthesia and surgical procedure and were randomly assigned for LepA or saline injection at reperfusion of the right ICA.

**5. Blinding**

All measurements of histological results and data analysis were performed by two investigators blinded to the identity of the samples.

**6. Outcome measures**

We analyzed brain tissue slides by histochemistry (H&E) assessing CA1 pyramidal cells for morphological features to determine cellular viability. We also used immunohistochemistry to assess staining for caspase 3, TUNEL, Iba1, pSTAT3 and pSmad2.

**7. Statistical methods**

When appropriate, the Kolmogorov–Smirnov normality test was used to determine normal distribution of the data, and the F-test or Brown-Forsythe test for determining homogeneity of variance. For normally distributed data with equal variance, we used one-way ANOVA or two-tailed unpaired Student t test. For normally distributed data with unequal variance, we used Kruskal–Wallis to compare experimental groups. For post hoc analysis, multiple comparisons were corrected using Dunn test or Tukey. The p-values smaller than 5% were considered significant. In all experiments, measurements were taken from distinct samples (different animals). All measured data are presented as means and include all individual values.

**8. Experimental animals**

Wild type C57BL/6 12 weeks old male mice, weighing 23.5-25gr.

**9. Experimental procedure**

All mice were anesthetized with isoflurane (2.5% for induction and 1.5% for maintenance via a nose cone). All subjects underwent bilateral CCA and ECA ligation. The right CCA was reperfused after 12 min ischemia, followed by instant bolus injection of either low dose LepA or saline into the right ICA through the ECA stump. The left common carotid artery remained ligated. Fifteen IR/LepA-injected and 14 IR/saline injected mice completed the experiment. Mice were euthanized on postoperative day 5, and formalin fixed brain samples were analyzed using H&E staining and immunohistochemistry for caspase 3, TUNEL, Iba1, pSTAT3 and pSmad2.

**10. Results**

Pyramidal cells in the CA1 region subjected to IR that received saline injection at reperfusion exhibited extensive cell death. Positive staining for apoptosis marker caspase 3, nuclear DNA fragmentation via TUNEL staining and augmented neuroinflammation (Iba1) were evident locally. We also demonstrated prevalent pSTAT3-positive and pSmad2-positive cells surrounding the stratum pyramidale. In contrast, pyramidal cells in the CA1 region of brains subjected to a similar insult and receiving selective LepA injection at reperfusion demonstrated pyramidal cell preservation (p<0.001). There were rare caspase 3-positive, seldom TUNEL-positive cells (p<0.05; p<0.001, respectively), and reduced density of activated microglia (p<0.001) in the CA1 region. pSTAT3 signal was undetected, and the presence of pSmad2 -positive cells surrounding the CA1 stratum pyramidale was greatly reduced (IR/LepA-injected versus IR/saline-injected, p<0.01.
